# Planting Patterns Affect the Differences in Growth and Its Responses to Nitrogen Forms and Levels Between Three Invasive and Their Respective Related Native Species

**DOI:** 10.3390/plants14121768

**Published:** 2025-06-10

**Authors:** Wei-Wei Feng, Kai Huang, Si-Miao Sun, Jian-Kun Sun, Ming Guan, Fa-Zhao Qi, Ming-Chao Liu, Bo Qu, Yu-Long Feng

**Affiliations:** 1 Liaoning Key Laboratory for Biological Invasions and Global Changes, College of Bioscience and Biotechnology, Shenyang Agricultural University, Shenyang 110866, China; shuiwutianxia@hotmail.com (W.-W.F.); kaihsyau@163.com (K.H.); sunsimiao@caas.cn (S.-M.S.); qfz_9708@163.com (F.-Z.Q.); lmc8866@syau.edu.cn (M.-C.L.); 1996500013@syau.edu.cn (B.Q.); 2 College of Ecology and Environment, Southwest Forestry University, Kunming 650233, China; 13998311092@163.com; 3 School of Life Sciences, Taizhou University, Taizhou 318000, China; guanmingtzc@163.com

**Keywords:** ammonium, invasive alien plants, mixed culture, monoculture, nitrate, nitrogen addition, nitrogen form preference

## Abstract

Global changes, such as atmospheric nitrogen deposition, can facilitate alien plant invasions, which are often attributed to the increase in soil nitrogen availability. However, few studies have considered the effects of global change-driven alterations in soil nitrogen forms, especially under conditions with interspecific competition. In this study, we first determined the differences in growth, biomass allocation, and photosynthesis under different nitrogen forms and addition levels between three noxious invasive species (*Xanthium strumarium*, *Ambrosia trifida*, and *Bidens frondosa*) and their respective related natives grown with and without interspecific competition and then assessed the interspecific difference in nitrogen form preference using the ^15^N labeling technique. Interspecific competition significantly decreased the positive responses of growth to nitrogen addition for all three natives, while increasing the responses for all three invaders, particularly under nitrate addition. When grown in competition, all invaders showed significant growth advantages over their related natives in most cases, and responded more positively to the addition of nitrate relative to ammonium, while the natives responded more positively to ammonium addition. These findings indicate that the invaders prefer nitrate, while the natives prefer ammonium. Consistently, the growth advantages are more pronounced for the invaders under nitrate relative to ammonium addition, indicating that nitrate-rich habitats may be more vulnerable to the invaders. When grown in monoculture, however, the growth advantage of the invaders became smaller or even disappeared. Nitrogen form preference also disappeared in *Siegesbeckia glabrescens* (native) and *Bidens frondosa* (invasive). Interestingly, the native plant *Xanthium sibiricum* showed significantly higher total biomass than its invasive congener under ammonium addition in both mixed and monoculture conditions. Our ^15^N labeling experiment showed that all six species preferred nitrate over ammonium, although this was not significant for two natives (*S. glabrescens* and *X. sibiricum*), which is not completely consistent with the results from our nitrogen addition experiment. Our results indicate that global change-driven alterations in soil nitrogen forms, particularly the shift from ammonium to nitrate, may facilitate alien plant invasions. Planting patterns significantly affect the responses of invasive and native species to nitrogen forms and addition levels, with mixed-culture experiments providing better insights into the invasiveness of alien species.

## 1. Introduction

Alien plant invasions are a significant ecological issue, causing substantial alterations in species composition, community structure, and ecosystem function [1,2,3]. Understanding the drivers of alien plant invasions is crucial not only for theoretical advancements but also for providing a scientific basis for predicting the distribution of invasive species and developing effective control strategies [4]. Anthropogenic atmospheric nitrogen deposition is a serious global environmental issue [5,6] and has been demonstrated to facilitate alien plant invasions and exacerbate their ecological damage [7,8,9]. For example, Lei et al. (2012) found that the noxious invasive plant *Eupatorium adenophorum* responds more strongly to nitrogen addition than its native congeners [8]. However, most related studies have focused on the effects of soil nitrogen levels, and the effects of nitrogen forms has received comparatively less attention [10,11]. Investigating how varying nitrogen forms affect alien plant invasions will provide a more comprehensive understanding of the mechanisms underlying the invasions.

The ratio of nitrate (NO_3_^−^) to ammonium (NH_4_^+^) in soil varies with environments and is always influenced by global change. NH_4_^+^ is generally the dominant nitrogen form in infertile, oxygen-limited, or acidic soils [12,13], while NO_3_^−^ is dominant in fertile, well-aerated, or alkaline soils [14,15]. The nitrogen forms in atmospheric deposition also fluctuate, and the proportion of nitrate increases gradually [16,17]. Human disturbances, particularly through agricultural practices, can enhance the activities of soil nitrifying bacteria, promoting soil nitrification and thus increasing NO_3_^−^ content [12,13,15,18,19]. For instance, Guo and Jia (2014) found that soil nitrification rates are higher in frequently disturbed agro-ecosystems than in forest ecosystems, which are positively correlated with soil pH and NO_3_^−^ levels [20]. Furthermore, excessive use of nitrogen fertilizer can also cause NO_3_^−^ (more mobile in soil than NH_4_^+^) to enter non-agro-ecosystems via runoff, elevating soil NO_3_^−^ availability and NO_3_^−^/NH_4_^+^ ratio [21]. Disturbed habitats are more susceptible to alien plant invasions than undisturbed or minimally disturbed habitats [22], which is often attributed to disturbance-driven increase in soil nutrient availability [23]. Besides the increased soil nitrogen availability, the disturbance-altered soil nitrogen forms may also contribute to alien plant invasions.

Numerous studies have demonstrated that plant species differ in their capacity to utilize soil NO_3_^−^ and NH_4_^+^ [10,24,25], and often exhibit preference for a specific nitrogen form, i.e., the uptake proportion of this form of nitrogen by plants is significantly higher than its proportion in soil [11,13,26]. For instance, several invasive plants, including *Bidens pilosa*, *Flaveria bidentis*, *Ipomoea cairica*, *Mikania micrantha*, *Wedelia trilobata*, and *Xanthium strumarium* all have been shown to prefer NO_3_^−^ [11,17,27]. However, *W. trilobata* is also found to prefer NH_4_^+^ under certain environmental conditions [28]. This discrepancy in nitrogen form preference may possibly be associated with the differences in experimental conditions, such as soil nitrogen availability. *Solidago canadensis* also shows a context-dependent nitrogen form preference: preference for NH_4_^+^ in farmlands and abandoned fields, but preference for NO_3_^−^ in roadsides. These results indicate that plant nitrogen form preference is influenced by numerous factors, including soil inorganic nitrogen pool and NO_3_^−^/NH_4_^+^ ratio [29]. Interspecific competition may alter soil nutrient dynamics and thus affect plant nitrogen form preference [30]. These findings suggest that global change-driven alterations in soil nitrogen forms and availability may affect future invasion potential and the geographic spread of invasive alien plants. Consequently, studying the differential responses of invasive and native plants to different nitrogen forms is crucial not only for revealing invasion mechanisms but also for developing effective management strategies for invasive plants in the context of global change.

In this study, three noxious invasive alien plants, *Ambrosia trifida* L., *Bidens frondosa* Buch.-Ham. ex Hook.f., and *Xanthium strumarium* L., were compared with their phylogenetically related native plants, *Siegesbeckia glabrescens* M., *B. biternata* L., and *X. sibiricum* Patrin ex Widder, respectively, in a common garden. *Ambrosia trifida* has no native congeners in China, and thus was compared with *S. glabrescens*, a native forb from the same tribe (Trib. Heliantheae Cass) as the invader. Related species are more comparable than distantly related ones as they share more similar life history and functional traits [8,31]. *Xanthium strumarium* was first found in Beijing in 1991, *A. trifida* in Liaoning in 1949, and *B. frondosa* in Jiangsu in 1926 [22]. In northeast China, *X. strumarium* and *B. frondosa* were first documented in Liaoning in 2007 and Heilongjiang in 1950, respectively. All the invaders are native to North America, and now are widespread across many provinces in China, causing severe environmental and socio-economic problems. All three natives often co-occur with their invasive relatives in Liaoning, northeast China.

First, we determined the differences in responses of growth, biomass allocation, and photosynthesis to NH_4_^+^ versus NO_3_^−^ addition between each invasive and its related native species grown in both mixed and monoculture. Second, we assessed interspecific difference in nitrogen form preference using the ^15^N isotope labeling technique. We hypothesize that (1) nitrogen addition increases growth more greatly for all invaders compared with their respective related natives; (2) the extents to which the invaders prefer NO_3_^−^ over NH_4_^+^ are greater than that for their related natives, and addition of nitrate relative to ammonium more greatly promotes growth of the invaders, increasing their growth advantages and thus facilitating their invasions; (3) the above-mentioned phenomena are more pronounced in mixed culture than in monoculture. By determining the responses of related invasive and native species to different nitrogen forms and addition levels under both mono- and mixed-cultures, our study will provide new insights into the mechanisms underlying alien plant invasions in the context of global change. Direct assessment of invasive—native differences in nitrogen form preference adds a unique dimension to the study, which is beneficial for interpreting the results of our nitrogen addition experiment.

## 2. Results

### 2.1. Total Biomass, Root to Shoot Ratio and Photosynthesis in Monoculture

**Figure 1:**
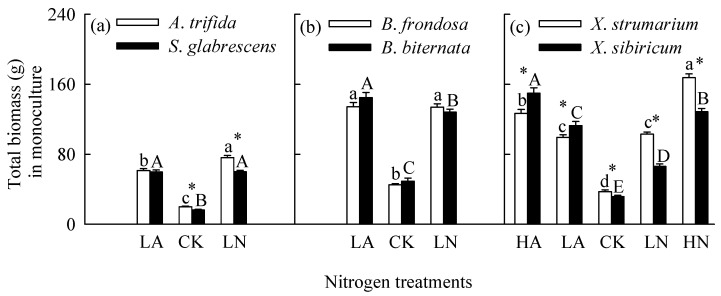
Total biomass of the invasive (open bars) and native (closed bars) species grown individually under different nitrogen forms and addition levels. CK, control; LA, low ammonium; LN, low nitrate; HA, high ammonium; HN, high nitrate. Panel (**a**), *Ambrosia trifida* vs. *Siegesbeckia glabrescens*; panel (**b**), *Bidens frondosa* vs. *B. biternata*; panel (**c**), *Xanthium strumarium* vs. *X. sibiricum*. Mean ± SE (*n* = 6). Different lower- and uppercase letters indicate significant differences among nitrogen treatments for the invasive and native species, respectively (*p* < 0.05; one-way ANOVA); * indicates significant difference between invasive and its related native species under the same nitrogen treatment (*p* < 0.05; independent samples *t*-test).

**Figure 2:**
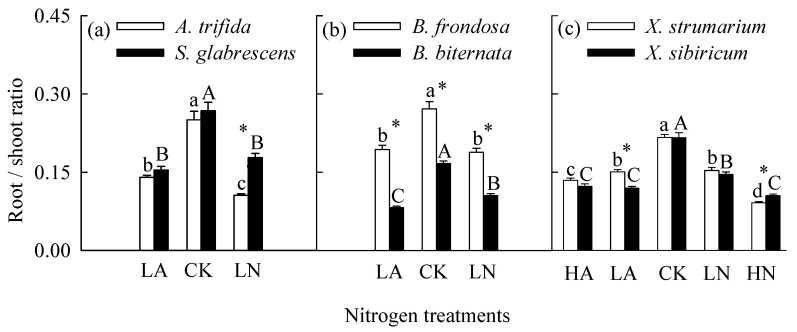
Root to shoot ratios of the invasive (open bars) and native (closed bars) species grown individually under different nitrogen forms and addition levels. CK, control; LA, low ammonium; LN, low nitrate; HA, high ammonium; HN, high nitrate. Panel (**a**), *Ambrosia trifida* vs. *Siegesbeckia glabrescens*; panel (**b**), *Bidens frondosa* vs. *B. biternata*; panel (**c**), *Xanthium strumarium* vs. *X. sibiricum*. Mean ± SE (*n* = 6). Different lower− and uppercase letters indicate significant differences among nitrogen treatments for the invasive and native species, respectively (*p* < 0.05; one-way ANOVA); * indicates significant difference between invasive and its related native species under the same nitrogen treatment (*p* < 0.05; independent samples *t*-test).

**Figure 3:**
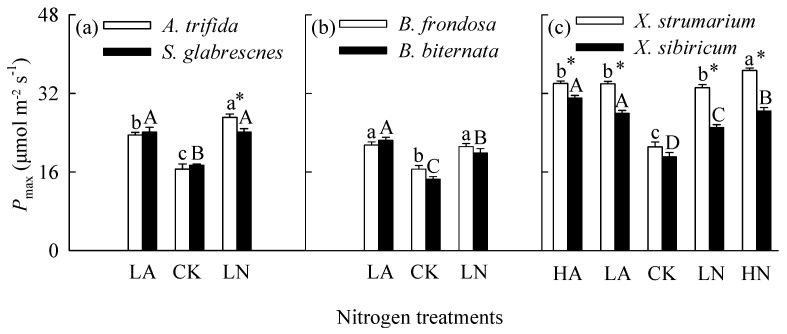
Light-saturated photosynthetic rates of the invasive (open bars) and native (closed bars) species grown individually under different nitrogen forms and addition levels. CK, control; LA, low ammonium addition; LN, low nitrate addition; HA, high ammonium addition; HN, high nitrate addition. Panel (**a**), *Ambrosia trifida* vs. *Siegesbeckia glabrescens*; panel (**b**), *Bidens frondosa* vs. *B. biternata*; panel (**c**), *Xanthium strumarium* vs. *X. sibiricum*. Mean ± SE (*n* = 6). Different lower− and uppercase letters indicate significant differences among nitrogen treatments for the invasive and native species (*p* < 0.05; one-way ANOVA); * indicates significant difference between invasive and its related native species under the same nitrogen treatment (*p* < 0.05; independent samples *t*-test).

**Figure 4:**
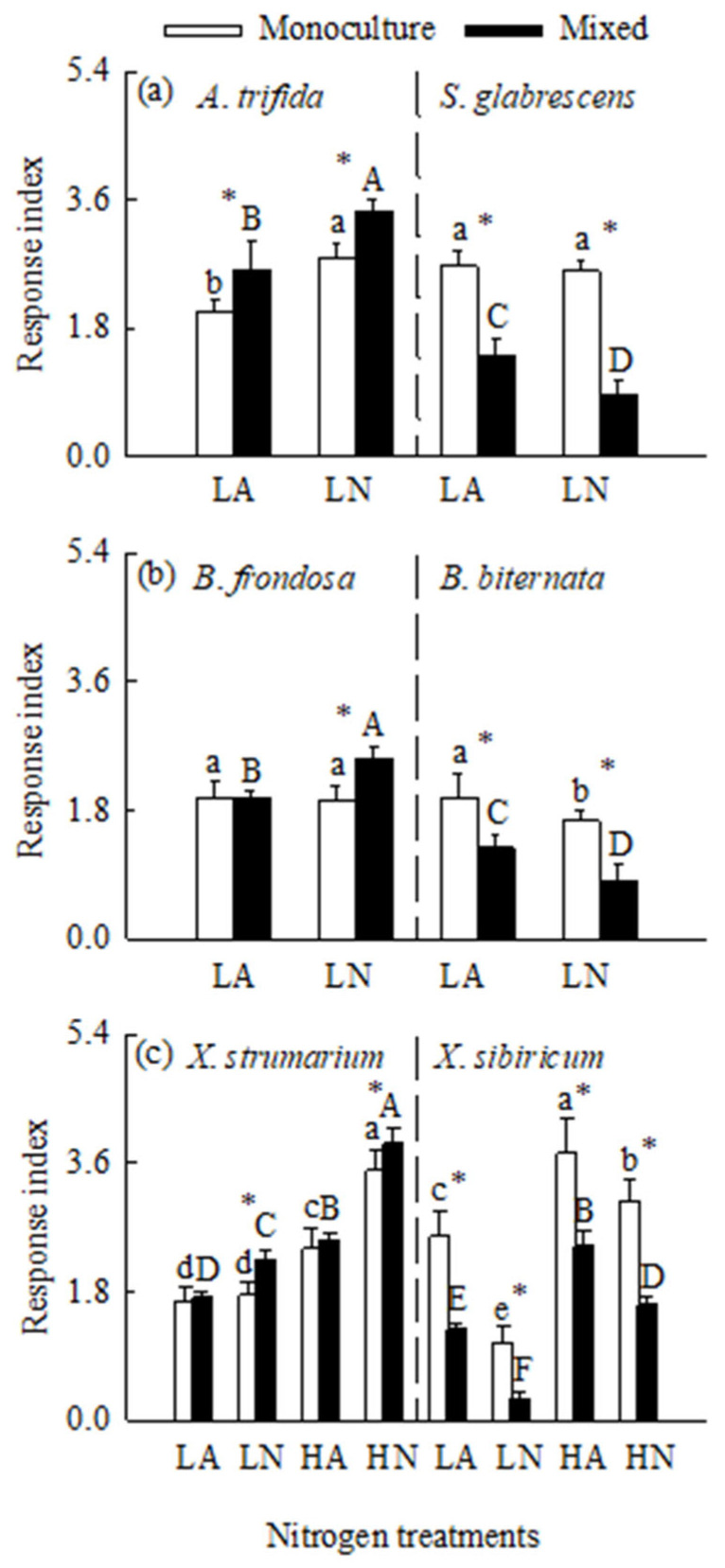
Response indices to different nitrogen forms and addition levels of the invasive and native species grown without (open bars) and with (closed bars) competition. LA, low ammonium; LN, low nitrate; HA, high ammonium; HN, high nitrate. Panel (**a**), *Ambrosia trifida* vs. *Siegesbeckia glabrescens*; panel (**b**), *Bidens frondosa* vs. *B. biternata*; panel (**c**), *Xanthium strumarium* vs. *X. sibiricum*. Mean ± SE (*n* = 6). Different lower− and uppercase letters indicate significant differences among the invasive and its related native species grown under different nitrogen treatments in the conditions without and with competition, respectively (*p* < 0.05; one-way ANOVA); * indicates significant difference between mixed and monoculture for the invasive (left) and native (right) species grown under the same nitrogen treatment (*p* < 0.05; independent samples *t*-test).

When grown in monoculture, nitrogen addition significantly increased total biomass and leaf light-saturated photosynthetic rate (*P*_max_), but decreased root to shoot ratio for all six species (Figure 1, Figure 2 and Figure 3; Table S1). For the invasive plants *A*. *trifida* and *X*. *strumarium* (except under low nitrogen treatments), the increases in total biomass and *P*_max_, and the decrease in root to shoot ratio were significantly greater under nitrate relative to ammonium treatments (Figure 1a,c, Figure 2a,c and Figure 3a,c; Table S2). However, the native plants *B*. *biternata* and *X*. *sibiricum* showed greater responses to the addition of ammonium relative to nitrate (Figure 1b,c, Figure 2b,c and Figure 3b,c). For *B*. *frondosa* (invasive) and *S*. *glabrescens* (native), nitrogen forms had no significant effects on these traits (Figure 1a,b, Figure 2a,b, and Figure 3a,b). Consistently, *A*. *trifida* and *X*. *strumarium* (except under low nitrogen addition levels) responded more strongly to nitrate addition compared with ammonium addition, *B*. *biternata* and *X*. *sibiricum* more strongly to ammonium addition, and *B*. *frondosa* and *S*. *glabrescens* responded similarly to different nitrogen forms (Figure 4; Table S3). Consistently, our ANOVA also showed that species and its interactions with nitrogen forms and/or addition levels significantly affected biomass response index for all species pairs (Table S3).

The invasive plant *A*. *trifida* had significantly higher total biomass (also in control treatment) and *P*_max_, but lower root to shoot ratio than *S*. *glabrescens* when grown in nitrate addition treatment, but not in ammonium addition treatment (Figure 1a, Figure 2a and Figure 3a; Table S2). For total biomass and *P*_max_, *B*. *frondosa* and *B. biternate* were similar in all nitrogen treatments, while root to shoot ratio was lower for the invader (Figure 1b, Figure 2b and Figure 3b; Tables S1 and S2). Total biomass was significantly higher for *X*. *strumarium* than *X. sibiricum* when grown in control and nitrate addition treatments, but lower in ammonium addition treatment (Figure 1c). This invader had higher *P*_max_ in all nitrogen treatments (not significant in control; Figure 3c; Tables S1 and S2). Root to shoot ratios of *X*. *strumarium* and *X. sibiricum* did not differ significantly under most conditions (Figure 2c).

### 2.2. Total Biomass in Mixed Culture

**Figure 5:**
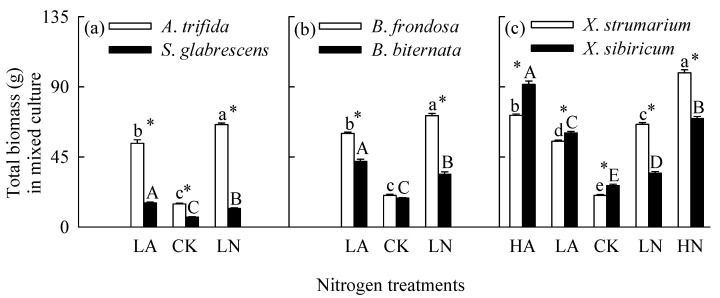
Total biomass of the invasive (open bars) and native (closed bars) species grown under different nitrogen forms and addition levels in mixed culture. CK, control; LA, low ammonium; LN, low nitrate; HA, high ammonium; HN, high nitrate. Panel (**a**), *Ambrosia trifida* vs. *Siegesbeckia glabrescens*; panel (**b**), *Bidens frondosa* vs. *B. biternata*; panel (**c**), *Xanthium strumarium* vs. *X. sibiricum*. Mean ± SE (*n* = 6). Different lower- and uppercase letters indicate significant differences among nitrogen treatments for the invasive and native species, respectively (*p* < 0.05; one-way ANOVA); * indicates significant difference between invasive and its related native species under the same nitrogen treatment (*p* < 0.05; independent samples *t*-test).

Interspecific competition greatly decreased total biomass for all six species (Figure 1 and Figure 5). Nitrogen addition also increased total biomass for all six species when grown in mixed culture (Figure 5; Table S1). Total biomass was significantly higher for all three invasive species, but lower for all three natives in nitrate relative to ammonium treatments, which were consistent with the significant interaction between species and nitrogen forms for all species pairs (Table S2). The invasives responded more greatly to nitrate relative to ammonium addition, while the natives more greatly to ammonium addition (Figure 4; Table S3). These results were not consistent with those for *B*. *frondosa* and *S*. *glabrescens* grown in monoculture. In addition, competition significantly decreased biomass responses to nitrogen addition of both forms for all three natives, while competition increased biomass response to nitrogen addition for *A*. *Trifida*, *B. frondasa* (in nitrate addition), and *X*. *strumarium* (in nitrate addition) (Figure 4). Consistently, species and its interactions with planting patterns or with nitrogen forms significantly affected biomass response index for all species pairs (Table S3).

In all nitrogen treatments, the invasive plants *A*. *Trifida* and *B. frondasa* (except in control) had significantly higher total biomass than their related natives, respectively, especially under nitrate addition treatments (Figure 5a,b, Tables S1 and S2). For *X*. *strumarium* compared with its native congener, total biomass was significantly higher in nitrate addition treatment, but lower in ammonium and control treatments (Figure 5c), consistent with the significant interaction between species and nitrogen forms (Table S2).

### 2.3. Uptake of Different Forms of Nitrogen

**Figure 6:**
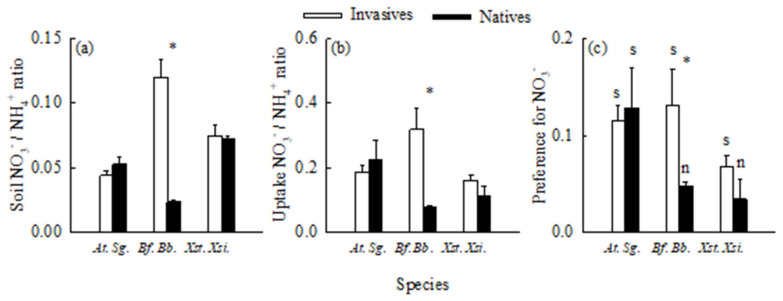
The ratios of nitrate nitrogen to ammonium nitrogen in soils (**a**), the ratios of nitrate nitrogen to ammonium nitrogen absorbed by roots (**b**), and the preference indices for nitrate (**c**) in the invasive (open bars) and native (closed bars) species. *At*., *Ambrosia trifida*; *Sg*., *Sigesbeckia glabrescens*; *Bf*., *Bidens frondosa*; *Bt*., *B*. *biternata*; *Xst*., *Xanthium strumarium*; *Xsi*., *X. sibiricum*. Mean ± SE (*n* = 5 for panel a; *n* = 3 for panels (**b**,**c**)). * indicates significant difference between invasive and its related native species (*p* < 0.05; independent samples *t*-test); s and n in panel (**c**) indicate that the preference were significant and non-significant (*p* < 0.05; independent samples *t*-test), respectively.

Ammonium was the dominant soil nitrogen form for all six species (Figure 6a). The ratios of soil nitrate nitrogen to ammonium nitrogen varied between 0.024 (*B. biternata*) and 0.120 (*B. frondosa*), with a mean of 0.065. Consistently, ammonium was the main nitrogen form absorbed by all six plants (Figure 6b). The uptake ratios of nitrate nitrogen to ammonium nitrogen varied from 0.076 (*B. biternata*) to 0.320 (*B. frondosa*), with a mean of 0.180. However, nitrate was the nitrogen form preferred by the six species (uptake proportion was higher than its proportion in soil), although the preference was not significant by *X. sibiricum* and *S. glabrescens* (Figure 6c).

The ratios of nitrogen from soil nitrate to ammonium, the ratios of nitrogen absorbed from nitrate to ammonium, and the preference for nitrate (*p* = 0.092) were all significantly higher for *B. frondosa* compared with its native congener, but not for *A. trifida* and *X. strumarium* compared with their related natives (Figure 6; Table S4).

## 3. Discussion

Consistent with our hypothesis, nitrogen addition significantly increased total biomass for the invasive plants *Ambrosia trifida*, *Bidens frondosa*, and *Xanthium strumarium*, as well as their related natives. Similar results were also found in numerous references [8,32,33]. However, our study further revealed that the effects of nitrogen addition were influenced by both nitrogen forms (nitrate vs. ammonium) and planting patterns (mixed vs. monoculture). This underscores the intricate interplay between soil nitrogen dynamics and plant community structure, where soil nitrogen forms and availability, along with planting patterns, interact to shape the growth responses of invasive and native species under different environmental conditions.

### 3.1. Effects of Competition on Responses to Nitrogen Addition for Invasive Versus Native Plants

As hypothesized, interspecific competition increased growth advantages for all three invasive species over their respective related natives in most cases. For example, competition either initiated (start from scratch; in two cases) or further increased (in three cases) growth advantages for the invasive plants *A. trifida* and *B. frondosa* over their respective related natives under all nitrogen treatments. Competition also led to further increases in growth advantage for the invasive plant *X. strumarium* over its native congener under nitrate addition treatments. These results align with the findings that invasive species tend to outperform natives in competitive environments due to their higher nitrogen uptake rates and use efficiencies [29,34]. When grown in competition, the invasive species may absorb most of the nitrogen added (and other nutrients), leaving less for their respective related natives, and thus exacerbating their growth advantage. Consistently, all invaders responded more greatly to nitrogen addition when grown in mixed versus monoculture, while the opposite was true for all natives. Our findings further showed that invasive species can more effectively exploit soil available nitrogen, particularly in mixed-culture conditions, giving a possible explanation for the stronger growth advantage of the invaders over their related natives when grown in mixed culture compared with monoculture.

### 3.2. Effects of Competition on Responses to Different Nitrogen Forms for Invasive Versus Native Plants

As expected, interspecific competition also influenced growth responses to different nitrogen forms for the invasive and native species. When grown in competition, all invaders responded more greatly to nitrate addition relative to ammonium addition (preferring nitrate), while the opposite was true for all natives (preferring ammonium). When grown in monoculture, however, the invasive plant *B. frondosa* and the native plant *Siegesbeckia glabrescens* no longer showed nitrogen form preference as they responded similarly to nitrate versus ammonium. Notably, the preferred nitrogen form (nitrate) for the six species was not consistent with the form that they absorbed most (ammonium). Furthermore, nitrogen form preference (responses to different nitrogen forms) for the six studied species grown in mixed culture was also not completely consistent with the results from our ^15^N labeling experiment (conducted in monoculture). Similarly, the effects of competition on nitrogen form preference were also found for the invasive plant *Microstegium vimineum*, which prefers nitrate in monoculture, but does not show a clear preference for either nitrogen form in mixed culture [10].

The observed discrepancy in nitrogen form preference between mixed and monoculture conditions could be attributed to competition-induced changes in soil nitrogen availability and the ratio of nitrate to ammonium, which have been demonstrated to affect plant nitrogen form preference [29,33]. In mixed cultures, interspecific competition may likely reduce soil nitrogen availability and the proportion of nitrate, as the invaders preferentially absorb nitrate.

Interestingly, for the six studied species, nitrogen form preferences based on our ^15^N labeling experiment (conducted in monoculture) were not entirely consistent with the results from our nitrogen addition experiment. This discrepancy may likely stem from the distinct methodological frameworks employed in these experiments. The ^15^N labeling approach directly measured nitrogen uptake, providing precise insights into plant preference for different nitrogen forms. In contrast, the nitrogen addition experiment assessed the overall growth responses, which were influenced by interspecific competition and other environmental factors. Our results highlight the complexity of nitrogen form preference in plants, as they are modulated not only by soil N forms and availability but also by interspecific competition.

### 3.3. Effects of Nitrogen Forms on Alien Plant Invasions

As expected, nitrogen forms significantly influenced the differences in growth and responses to nitrogen addition between the three invasive species and their respective related natives. Growth advantages were higher for the invaders over their related natives under nitrate relative to ammonium nitrogen addition when grown in mixed culture, indicating that habitats with higher nitrate availability or proportions are more vulnerable to invasive plants. Consistently, invasive plants are frequently found in disturbed habitats such as roadsides, riversides, wastelands, farmlands, and secondary forests [22], where soil nitrification rates are generally high, leading to increased soil nitrate availability [15,19]. These findings underscore that global change, for example atmospheric nitrogen deposition, human disturbance on natural ecosystems, and agricultural non-point source pollution, may exacerbate alien plant invasions by altering soil nitrogen dynamics.

For the invasive plant *X. strumarium*, preference for nitrate over ammonium was also found in other studies with controlled experiments [17]. This invader may also prefer nitrate in the field, as judged by its higher pH and net nitrification rates in rhizosphere soils compared with its native congener. It is well known that invasive plants with a preference for nitrate can increase soil pH, enhancing nitrification [12,35]. For *X. strumarium*, the ability to absorb and assimilate nitrate relative to ammonium is higher, while the opposite is true for *X. sibiricum* [20]. A growing number of studies have found that invasive plants prefer nitrate, such as *Berberis thunbergii*, *Microstegium vimineum*, *Avena barbata*, *Bromus hordeaceous*, *B. tectorum*, *Amaranthus retroflexus*, *Bidens pilosa*, *Ipomoea cairica*, and *Mikania micrantha* [10,18,27,36,37,38,39]. This preference may enhance the fitness and dominance of invasive plants within communities with nitrate as the dominant form of soil nitrogen. Consistently, alien plants often prefer to invade disturbed habitats, where nitrate is generally the main soil nitrogen source. Recently, Chang et al. (2025) found that invasive species benefit from matching their preferential nitrogen uptake with soil nitrogen dynamics [40]. Guan et al. (2025) further found that the preference of numerous plants for major forms of soil nitrogen contributes to their nitrogen uptake and therefore to their dominance within three communities [41]. In addition, *X. strumarium* had higher photosynthesis and a lower root to shoot ratio under nitrate relative to ammonium addition, also contributing to its higher total biomass.

## 4. Materials and Methods

### 4.1. Study Site and Seed Collection

This study was conducted at a common garden in the experimental base (41°50′ N, 123°34′ E; 59 m asl) of Shenyang Agricultural University located in Shenyang, Liaoning Province, northeast China. This site has a temperate continental monsoon climate, with hot, moist summers and cold, dry winters. The mean annual temperature is 8.1 °C, and the mean annual precipitation is 721.9 mm, with most rainfall occurring in July and August.

Seeds of each of the six studied species (see the Section 1) were collected in the autumn of the year before the experiments from more than ten individuals (spaced > 20 m apart) in a disturbed habitat near the Hunhe River (41°49′ N, 123°34′ E; 28 m asl) in Fushun, Liaoning Province. The seeds were stored at 4 °C until used.

### 4.2. Experiment I: Responses to Nitrogen Forms and Addition Levels

#### 4.2.1. Seed Germination and Seedling Transplant

In mid-April, full and healthy seeds of each species were soaked separately in distilled water for 30 min and then placed in petri dishes with wet filter papers for germination. Germination was conducted in growth chambers with 25/22 °C (day/night) and 108 μmol m^−2^ s^−1^ light intensity (12/12 h photoperiod). For *X*. *strumarium* and *X*. *sibiricum*, only the lower seeds were used due to their larger size and shorter dormancy time compared with the upper seeds [42,43]. Before germination, the seeds had been stratified in wet sand at 4 °C for two months.

After germination, the seeds were transferred into seedling trays filled with a substrate mixture of forest topsoil (0–10 cm) and medium-grained sand (7:3 ratio) and grown in a greenhouse. Similarly sized seedlings (≈10 cm tall) for each invasive-native species pair were transplanted into pots (top diameter × bottom diameter × height: 30 × 25 × 22 cm). Two planting treatments were applied for each species pair: monoculture (one seedling per pot) and mixed culture (one invasive and one native seedling per pot, spaced 5 cm apart). The soil was collected from a secondary forest in Shenyang, Liaoning Province, air-dried, and passed through a 5 mm sieve. The sand was collected from Hunhe in Shenyang. For the growth substrate, pH was 7.32. The contents of organic matter, total nitrogen, total phosphorus, total potassium, available nitrogen, available phosphorus, and available potassium were 5.57 mg g^−1^, 0.31 mg g^−1^, 0.57 mg g^−1^, 13.6 mg g^−1^, 20.99 μg g^−1^, 5.99 μg g^−1^, and 55.69 μg g^−1^, respectively. Soil organic matter, total nitrogen, and available nitrogen were extremely poor for the substrate based on the nutrient classification standard of the second national soil survey [44].

#### 4.2.2. Nitrogen Treatments

One week after transplantation, the seedlings of all six studied species were treated with ammonium addition (40 mmol NH_4_H_2_PO_4_ and 40 mmol KH_2_PO_4_ per pot), nitrate addition (40 mmol KNO_3_ and 80 mmol H_3_PO_4_), and no nitrogen addition control (12 replicates per treatment). The nutrients were evenly applied four times for nitrogen addition treatments, at intervals of 10 d. Before application, a specified amount of nutrients was dissolved in water and then applied. For control, an equal amount of water was added only. To inhibit potential transformation of ammonium to nitrate, 0.8 mmol thiourea was added to each pot [45]. This amount of nitrogen (≈8 g m^−2^) was determined through our preliminary experiment, which was also within the nitrogen addition rates used in the literature [46]. Because the soil was extremely poor, the amount of nitrogen added was higher than that from atmospheric deposition. Higher levels of ammonium (80 mmol NH_4_H_2_PO_4_ and 80 mmol KH_2_PO_4_) and nitrate (80 mmol KNO_3_ and 160 mmol H_3_PO_4_) were also applied for the seedlings of *X. strumarium* and *X. sibiricum*, but not for those of the other four species. It has been found that *Ambrosia trifida* and *B*. *frondosa* respond slightly to high nitrogen levels [47,48].

#### 4.2.3. Measurements

About 50 d after seedling transplantation, light-saturated photosynthetic rates (*P*_max_) were measured for each species grown in monoculture using a Li-6400 Portable Photosynthesis Meter (Li-Cor, Lincoln, Nebraska, USA). Light intensity on leaf surface, relative humidity in leaf chamber, CO_2_ concentration in reference chamber, and leaf temperature were controlled at 1500 μmol m^−2^ s^−1^, 50%, 380 μmol mol^−1^, and 25 °C, respectively. Then, six individuals per species per nitrogen and competition treatment were randomly chosen and harvested. Each individual was separated into shoots and roots, dried at 60 °C for 72 h, and weighed. Total biomass was calculated as the sum of root and shoot biomass; root to shoot ratio was determined as the ratio between root and shoot biomass; the response index to nitrogen addition was calculated as the relative change in total biomass: (total biomass under nitrogen addition—mean total biomass under control)/mean total biomass under control.

### 4.3. Experiment II: Uptake of Different Forms of Nitrogen

#### 4.3.1. Preparation of Seedlings

The seedlings of the six species were prepared using a similar method to that described in Experiment I. However, smaller pots (top diameter × bottom diameter × height: 15 cm × 10 cm × 12 cm), less substrate (1.5 kg), and only monoculture (one seedling per pot) were used. In total, 180 pots were prepared (6 species × 30 replicates). The pots for each species pair were randomly arranged in a block within the common garden, spaced 60 cm apart, while blocks for different species pairs were spaced 2 m apart. The pots were watered daily and weeded when necessary.

#### 4.3.2. Nitrogen Stable Isotope Labeling, Sampling and Measurements

Forty-five days after seedling transplantation, when the plants grew at vigorous vegetative stage, the ^15^N isotope labeling method was used to quantify plant uptake of ammonium and nitrate. Nine individuals of each species with similar size were selected for three treatments: ^15^N-ammonium labeling ((^15^NH_4_)_2_SO_4_), ^15^N-nitrate labeling (K^15^NO_3_), and control (distilled water), with three replicates for each treatment. Around each seedling (*r* = 3.0 cm), 45 mL of 4.76 mmol nitrogen L^−1^ labeling solution (^15^N > 99%) or distilled water was injected into the soil (0–10 cm), based on preliminary experiments. For details, refer to Guan et al. (2023) [29]. This method ensured even dispersion of the injected solution within the pots, and the content of the ^15^N added was 2.1 μg g^−1^ dry soil.

Shoots and roots of the labeled and control plants were collected after 2 h of ^15^N labeling. The roots were immersed into a 0.5 mmol L^−1^ CaCl_2_ solution for 30 min to remove the ^15^N adhered to root surface [35] and then rinsed twice with deionized water. The shoots and roots from the same plant were dried at 60 °C for 72 h and then weighed. Both parts were mixed, ground into powder, and analyzed for total nitrogen content and ^15^N atom% excess using an elemental analysis-isotope ratio mass spectrometry (Flash 2000HT + Delta V Advantage, Thermo, Germany).

For each species, rhizosphere soil from each control individual was collected using the method described by Zhao et al. (2020) and sieved through a 2 mm mesh [3]. Soil ammonium and nitrate were extracted with 2 mol L^−1^ KCl, and quantified using an Automated Chemistry Analyzer (AA3, Seal, Germany). For each labeled individual of each species, rhizosphere soil content of the nitrogen with a specific form was calculated as the sum of the mean value of the measured contents for the control individuals and the amount of ^15^N added into soil (2.1 μg g^−1^). The ratios of nitrate to ammonium were also calculated.

#### 4.3.3. Calculations

For each labeled individual, the uptake of the labeled ^15^N from a specific form (^15^NH_4_^+^ or ^15^NO_3_^−^) was calculated using their ^15^N atom% excess (APE), total biomass (shoots and roots), and total N contents. The actual N uptake (including both ^14^N and ^15^N) was calculated for each labeled individual based on the uptake of the labeled ^15^N and the ratio of the labeled ^15^N to nitrogen naturally occurring in soil. This method assumes that ^14^N/^15^N isotope fractionation is negligible when plants are exposed to a low concentration of labeled nitrogen [29]. The uptake rate of each nitrogen form was then determined using the amount of N absorbed by each labeled individual, its root biomass, and labeling duration (2 h). Finally, nitrogen form preference was calculated for each labeled individual as the difference between plant uptake proportion of a given nitrogen form and its corresponding proportion in the soil. A positive value indicates a preference for that nitrogen form, whereas a negative value suggests preference for the other inorganic nitrogen form. For details, refer to Guan et al. (2023) and Sun et al. (2025) [29,33].

### 4.4. Statistical Analyses

Two-way analysis of variance (ANOVA) was used to test effects of species, nitrogen forms or addition levels, and their interaction on total biomass, root to shoot ratios, and *P*_max_ for each invasive and native species pair grown in mono- or mixed culture. The effects of nitrogen forms or addition levels were tested separately, as there are no nitrate and ammonium treatments in control (adding water only). The data in control were not included when testing the effects of species, nitrogen forms, and their interaction, and nitrogen addition treatments with nitrate and ammonium were not distinguished at the same nitrogen addition level when testing the effects of species, nitrogen addition levels, and their interaction. Three- and four-way ANOVA were used to test the effects of species, planting patterns, nitrogen forms, and addition levels (for *Xanthium* species pair only), and their interactions on biomass response index for each invasive and native species pair grown in mono- and mixed cultures.

One-way ANOVA was used to test the difference in each variable among nitrogen treatments for each species, the difference among invasive and its related native species under different nitrogen treatments in the same planting pattern (mixed or monoculture; for biomass response index only), and that between each invasive and its related native species in each variable measured in Experiment 2. Independent samples *t*-test was applied to compare the difference between each invasive and its related native species under each nitrogen treatment, the difference between mixed and monocultures for each invasive and native species under the same nitrogen treatment, and that between preference for nitrate and zero for each species (*p* < 0.05 indicates significant preference for nitrate). Data were transformed when necessary to meet the requirements of ANOVA. All analyses were performed using SPSS 20.0 version (SPSS Inc., Chicago, IL, USA).

## 5. Conclusions

Our results show that nitrogen forms significantly affect invasions of alien plant species besides soil N availability, and the effects of soil nitrogen availability are further shaped by nitrogen forms and planting patterns (mixed versus monoculture), with mixed-culture experiments proving a more comprehensive understanding of alien plant invasions. When grown in competition with their respective related native species, the invasive plants *A. trifida*, *B. frondosa*, and *X. strumarium* responded more positively to the addition of nitrate relative to ammonium nitrogen, while the natives responded more strongly to ammonium, indicating that the invaders prefer nitrate, while the natives prefer ammonium. Growth advantages of the invaders over their related natives were greater under addition of nitrate relative to ammonium nitrogen, indicating that nitrate-rich habitats may be more vulnerable to invaders. Our results indicate that global change-driven alterations in soil nitrogen forms may facilitate alien plant invasions, highlighting the importance of considering soil nitrogen forms when studying effects of global change such as atmospheric nitrogen deposition and human disturbance on invasions of introduced species. Our study also underscores the importance of considering interspecific interactions when studying invasion mechanisms and impacts in the context of global change.

## Data Availability

The original contributions presented in the study are included in the article/Supplementary Material. Further inquiries can be directed to the corresponding authors.

## Supplementary Materials

The following supporting information can be downloaded at https://www.mdpi.com/article/10.3390/plants14121768/s1. Table S1: Effects of species, nitrogen addition levels, and their interaction on total biomass, light-saturated photosynthetic rates, and root to shoot ratios for each invasive and native species pair; Table S2: Effects of species, nitrogen forms, and their interaction on total biomass, light-saturated photosynthetic rates, and root to shoot ratios for each invasive and native species pair; Table S3: Effects of species, planting patterns, nitrogen forms and addition levels (for *Xanthium* species pair only), and their interactions on biomass response index for each invasive and native species pair; Table S4: Effects of species on the ratios of nitrate nitrogen to ammonium nitrogen in soil, plant uptake ratios of nitrate nitrogen to ammonium nitrogen, and plant preference for nitrate for each invasive and native species pair.
