# Supplementary material for: Planting Patterns Affect the Differences in Growth and Its Responses to Nitrogen Forms and Levels Between Three Invasive and Their Respective Related Native Species
Source: Plants (Basel). 2025 Jun 10;14(12):1768. doi: 10.3390/plants14121768 (PMC12197312; doi:10.3390/plants14121768)
Supplement: Supplementary file 1 [file plants-14-01768-s001.zip › plants-3619805-supplementary.pdf]

Table S1 Effects of species ($n$ = 2), nitrogen addition levels ($n$ = 2), and their interaction on total biomass, light-saturated photosynthetic rates ($P_{max}$, μmol $CO_2$ $m^{-2}$ $s^{-1}$), and root to shoot ratios for each invasive and native species pair grown in mono- or mixed culture

| Source of variation | | Total biomass in monoculture | | | $P_{max}$ in monoculture | | | Root/Shoot in monoculture | | | Total biomass in mixed culture | | |
|---|---|---|---|---|---|---|---|---|---|---|---|---|---|
| | df | *MS* | *F*-value | *P-value* | *MS* | *F*-value | *P-value* | *MS* | *F*-value | *P-value* | *MS* | *F*-value | *P-value* |
| ***Ambrosia trifida* vs *Sigesbeckia glabrescens*** | | | | | | | | | | | | | |
| Species | 1 | 329.389 | 10.868 | **0.002** | 3.728 | 1.028 | 0.318 | 0.007 | 9.159 | **0.005** | 5945.453 | 270.847 | **< 0.001** |
| Nitrogen levels | 1 | 16541.986 | 545.782 | **< 0.001** | 497.111 | 137.012 | **< 0.001** | 0.105 | 131.54 | **< 0.001** | 5458.074 | 248.644 | **< 0.001** |
| Species × Nitrogen levels | 1 | 57.961 | 1.912 | 0.176 | 0.048 | 0.013 | 0.909 | 0.001 | 1.595 | 0.216 | 2833.311 | 129.072 | **< 0.001** |
| Standard error | 32 | 30.309 | | | 3.628 | | | 0.001 | | | 21.951 | | |
| ***Bidens frondosa* vs *B. biternata*** | | | | | | | | | | | | | |
| Species | 1 | 105.149 | 0.874 | 0.357 | 0.48 | 0.183 | 0.672 | 0.082 | 209.678 | **< 0.001** | 1731.367 | 67.109 | **< 0.001** |
| Nitrogen levels | 1 | 62932.65 | 522.983 | **< 0.001** | 372.317 | 141.601 | **< 0.001** | 0.047 | 120.962 | **< 0.001** | 8431.161 | 326.796 | **< 0.001** |
| Species × Nitrogen levels | 1 | 0.129 | 0.001 | 0.974 | 41.697 | 15.858 | **< 0.001** | 0 | 0.276 | 0.603 | 1361.985 | 52.791 | **< 0.001** |
| Standard error | 32 | 120.334 | | | 2.629 | | | 0 | | | 25.799 | | |
| ***Xanthium italicum* vs *X. sibiricum*** | | | | | | | | | | | | | |
| Species | 1 | 970.345 | 3.04 | ***0.087*** | 132.122 | 14.594 | **< 0.001** | 0.001 | 1.734 | 0.194 | 185.74 | 1.548 | 0.219 |
| Nitrogen levels | 2 | 48223.499 | 151.073 | **< 0.001** | 611.229 | 67.514 | **< 0.001** | 0.043 | 139.871 | **< 0.001** | 14683.659 | 122.371 | **< 0.001** |
| Species × Nitrogen levels | 2 | 41.313 | 0.129 | 0.879 | 30.341 | 3.351 | **< 0.001** | 0.001 | 2.38 | 0.102 | 392.699 | 3.273 | **0.046** |
| Standard error | 54 | 319.207 | | | 9.053 | | | 0 | | | 119.993 | | |

Note: *df*, degrees of freedom; *MS*, mean square. *P*-values, < 0.05, are showed in bold; and those, < 0.1 > 0.05, are showed in bold and italic. Nitrogen addition treatments with nitrate and ammonium were not distinguished at the same nitrogen addition level in the two-way ANOVA.

Table S2 Effects of species ($n$ = 2), nitrogen forms ($n$ = 2), and their interaction on total biomass, light-saturated photosynthetic rates ($P_{max}$, μmol $CO_2$ $m^{-2}$ $s^{-1}$), and root to shoot ratios for each invasive and native species pair grown in mono- or mixed culture

| Source of variation | df | Total biomass in monoculture | | | $P_{max}$ in monoculture | | | Root/Shoot in monoculture | | | Total biomass in mixed culture | | |
|---|---|---|---|---|---|---|---|---|---|---|---|---|---|
| | | *MS* | *F*-value | *P-value* | *MS* | *F*-value | *P-value* | *MS* | *F*-value | *P-value* | *MS* | *F*-value | *P-value* |
| ***Ambrosia trifida* vs *Sigesbeckia glabrescens*** | | | | | | | | | | | | | |
| Species | 1 | 497.77 | 40.907 | **< 0.001** | 2.198 | 0.704 | 0.411 | 0.011 | 55.285 | **< 0.001** | 12740.53 | 1195.708 | **< 0.001** |
| Nitrogen forms | 1 | 299.768 | 24.635 | **< 0.001** | 15.731 | 5.038 | **0.036** | 0 | 0.857 | 0.366 | 108.898 | 10.22 | **0.005** |
| Species × Nitrogen forms | 1 | 394.633 | 32.431 | **< 0.001** | 2.652 | 0.849 | 0.368 | 0.005 | 24.797 | **< 0.001** | 367.957 | 34.533 | **< 0.001** |
| Standard error | 32 | 12.168 | | | 3.122 | | | 0 | | | 10.655 | | |
| ***Bidens frondosa* vs *B. biternata*** | | | | | | | | | | | | | |
| Species | 1 | 84.488 | 0.619 | 0.441 | 38.345 | 13.127 | **0.002** | 0.057 | 268.965 | **< 0.001** | 4623.428 | 431.111 | **< 0.001** |
| Nitrogen forms | 1 | 374.855 | 2.747 | 0.113 | 0.735 | 0.252 | 0.621 | 0 | 2.186 | 0.155 | 14.03 | 1.308 | 0.266 |
| Species × Nitrogen forms | 1 | 349.073 | 2.558 | 0.125 | 0.795 | 0.272 | 0.608 | 0.001 | 5.728 | **0.027** | 577.515 | 53.85 | **< 0.001** |
| Standard error | 32 | 136.445 | | | 2.921 | | | 0 | | | 10.724 | | |
| ***Xanthium italicum* vs *X. sibiricum*** | | | | | | | | | | | | | |
| Species | 1 | 1167.917 | 1.238 | 0.272 | 163.373 | 11.635 | **0.001** | 0.001 | 2.037 | 0.161 | 950.458 | 3.746 | ***0.059*** |
| Nitrogen forms | 2 | 2949.878 | 3.127 | ***0.084*** | 66.03 | 4.703 | **0.036** | 0.001 | 1.574 | 0.216 | 67.339 | 0.265 | 0.609 |
| Species × Nitrogen forms | 2 | 104.932 | 0.111 | 0.74 | 20.59 | 1.466 | 0.232 | 0.002 | 3.69 | ***0.061*** | 5499.094 | 21.675 | **< 0.001** |
| Standard error | 54 | 943.478 | | | 14.041 | | | 0 | | | 253.702 | | |

Note: *df*, degrees of freedom; *MS*, mean square. *P*-values, < 0.05, are showed in bold; and those, < 0.1 > 0.05, are showed in bold and italic. The data in control were not included in the two-way ANOVA, as there were no nitrate and ammonium treatments in control (adding water only).

Table S3 Effects of species ($n$ = 2), planting patterns ($n$ = 2), nitrogen forms ($n$ = 2) and addition levels ($n$ = 2; for *Xanthium* species pair only), and their interactions on biomass response index for each invasive and native species pair grown in mono- and mixed cultures

| Source of variation | Biomass response index | | | |
|---|---|---|---|---|
| | *df* | *MS* | *F*-value | *P-value* |
| ***Ambrosia trifida* vs *Sigesbeckia glabrescens*** | | | | |
| Species | 1 | 8.325 | 163.283 | **< 0.001** |
| Planting patterns | 1 | 2.23 | 43.738 | **< 0.001** |
| Nitrogen forms | 1 | 0.679 | 13.322 | **0.001** |
| Species × Planting patterns | 1 | 13.367 | 262.189 | **< 0.001** |
| Species × Nitrogen forms | 1 | 3.647 | 71.53 | **< 0.001** |
| Planting patterns × Nitrogen forms | 1 | 0.146 | 2.869 | ***0.098*** |
| Species × Planting patterns × Nitrogen forms | 1 | 0.231 | 4.532 | **0.039** |
| Standard error | 40 | 0.051 | | |
| ***Bidens frondosa* vs *B. biternata*** | | | | |
| Species | 1 | 5.365 | 119.116 | **< 0.001** |
| Planting patterns | 1 | 0.657 | 14.581 | **< 0.001** |
| Nitrogen forms | 1 | 0.033 | 0.727 | 0.399 |
| Species × Planting patterns | 1 | 3.437 | 76.319 | **< 0.001** |
| Species × Nitrogen forms | 1 | 1.302 | 28.913 | **< 0.001** |
| Planting patterns × Nitrogen forms | 1 | 0.144 | 3.193 | ***0.082*** |
| Species × Planting patterns × Nitrogen forms | 1 | 0.363 | 8.049 | **0.007** |
| Standard error | 40 | 0.045 | | |
| ***Xanthium italicum* vs *X. sibiricum*** | | | | |
| Species | 1 | 4.855 | 85.731 | **< 0.001** |
| Planting patterns | 1 | 5.483 | 96.817 | **< 0.001** |
| Nitrogen levels | 1 | 41.749 | 737.195 | **< 0.001** |
| Nitrogen forms | 1 | 2.874 | 50.744 | **< 0.001** |
| Species × Planting patterns | 1 | 13.111 | 231.511 | **< 0.001** |
| Species × Nitrogen levels | 1 | 0.192 | 3.395 | ***0.069*** |
| Species × Nitrogen forms | 1 | 0.276 | 4.876 | **0.03** |
| Planting patterns × Nitrogen levels | 1 | 0.176 | 3.104 | ***0.082*** |
| Planting patterns × Nitrogen forms | 1 | 0.268 | 4.725 | **0.033** |
| Nitrogen levels × Nitrogen forms | 1 | 0.283 | 4.999 | **0.028** |
| Species × Planting patterns × Nitrogen levels | 1 | 0.156 | 2.756 | 0.101 |
| Species × Planting patterns × Nitrogen forms | 1 | 0.083 | 1.463 | 0.23 |
| Species × Nitrogen levels × Nitrogen forms | 1 | 18.394 | 324.793 | **< 0.001** |
| Planting patterns × Nitrogen levels × Nitrogen forms | 1 | 0.414 | 7.304 | **0.008** |
| Species × Planting patterns × Nitrogen levels × Nitrogen forms | 1 | 0.038 | 0.668 | 0.416 |
| Standard error | 80 | 0.057 | | |

Note: *df*, degrees of freedom; *MS*, mean square. *P*-values, < 0.05, are showed in bold; and those, < 0.1 > 0.05, are showed in bold and italic.

Table S4 Effects of species ($n$ = 2) on the ratios of nitrate nitrogen to ammonium nitrogen in soil, plant uptake ratios of nitrate nitrogen to ammonium nitrogen, and plant preference for nitrate for each invasive and native species pair

| Source of variation | Soil $NO_3^-/NH_4^+$ ratio | | | | Uptake $NO_3^-/NH_4^+$ ratio | | | | Preference for $NO_3^-$ | | | |
|---|---|---|---|---|---|---|---|---|---|---|---|---|
| | *df* | *MS* | *F*-value | *P-value* | *df* | *MS* | *F*-value | *P-value* | *df* | *MS* | *F*-value | *P-value* |
| ***Ambrosia trifida* vs *Sigesbeckia glabrescens*** | | | | | | | | | | | | |
| Between groups | 1 | 0 | 1.658 | 0.234 | 1 | 0.002 | 0.287 | 0.62 | 1 | 0 | 0.085 | 0.785 |
| Within groups | 8 | 0 | | | 4 | 0.007 | | | 4 | 0.003 | | |
| Total | 9 | | | | 5 | | | | 5 | | | |
| ***Bidens frondosa* vs *B. biternata*** | | | | | | | | | | | | |
| Between groups | 1 | 0.023 | 53.034 | **< 0.001** | 1 | 0.089 | 13.25 | **0.022** | 1 | 0.011 | 4.872 | ***0.092*** |
| Within groups | 8 | 0 | | | 4 | 0.007 | | | 4 | 0.002 | | |
| Total | 9 | | | | 5 | | | | 5 | | | |
| ***Xanthium italicum* vs *X. sibiricum*** | | | | | | | | | | | | |
| Between groups | 1 | 0 | 0.108 | 0.751 | 1 | 0.003 | 2.244 | 0.209 | 1 | 0.002 | 1.994 | 0.231 |
| Within groups | 8 | 0 | | | 4 | 0.001 | | | 4 | 0.001 | | |
| Total | 9 | | | | 5 | | | | 5 | | | |

Note: *df*, degrees of freedom; *MS*, mean square. *P*-values, < 0.05, are showed in bold; and those, < 0.1 > 0.05, are showed in bold and italic.
